# Liver organoids in domestic animals: an expected promise for metabolic studies

**DOI:** 10.1186/s13567-021-00916-y

**Published:** 2021-03-18

**Authors:** Camille Baquerre, Guillaume Montillet, Bertrand Pain

**Affiliations:** Univ Lyon, Université Lyon 1, INSERM, INRAE, Stem Cell and Brain Research Institute, U1208, USC1361, 69500 Bron, France

**Keywords:** Liver organoids, Pluripotent stem cells, Mammals, Birds, Domestic species

## Abstract

The liver is one of the most important organs, both in terms of the different metabolic processes (energy, lipid, ferric, uric, etc.) and of its central role in the processes of detoxification of substances of food origin or noxious substances (alcohol, drugs, antibiotics, etc.). The development of a relevant model that reproduces some of the functions of this tissue has become a challenge, in particular for human medicine. Thus, in recent years, most studies aimed at producing hepatocytes in vitro with the goal of developing hepatic 3D structures have been carried out in the human model. However, the tools and protocols developed using this unique model can also be considered to address physiological questions specific to this tissue in other species, such as the pig, chicken, and duck. Different strategies are presently being considered to carry out in vitro studies of the hepatic metabolism of these agronomic species.

## Introduction

The liver is a multifunctional organ that is central in controlling the metabolism of carbohydrates (conversion of glucose to glycogen), lipids (production of cholesterol and associated proteins), and amino acids (by regulating their circulating levels); in the synthesis of certain essential proteins (albumin, coagulation factors, etc.); in regulating the levels of circulating iron; and in converting ammonia to urea and bile by capturing bile acids. The liver also plays a key role in the detoxification of exogenous substances, such as antibiotics, alcohol, drugs, or other toxic substances. The multiple physiological functions of this organ are mainly ensured by hepatocytes, which constitute more than 80% of the cells of the liver and are organized in lobules. Other morphotypes are present in the liver, such as cholangiocytes in the bile ducts, endothelial cells, Kupffer cells (which are considered as hepatic macrophages) and hepatic stellate cells (which are resident lipid-storing cells) [1, 2]. During development, the hepatic outline appears on the ventral side of the distal end of the anterior intestine and results from complex interactions between mesoderm and endoderm cells at the intra- and extraembryonic junction of the yolk sac. Certain growth factors, such as Fibroblast growth factor (FGF), Bone morphogenetic proteins (BMPs), WNT signaling and retinoids allow the cellular signaling that is necessary for this differentiation of the hepatic lineage during development with a rapid dynamic [3–5].

Hepatocytes are extremely polarized epithelial cells that are organized into lobules. Lobules are anatomo-physiological structures that are more or less well defined morphologically according to animal species, with large disparities observed between humans and pigs including fibrotic bridges between periportal zones [6]. The basolateral side of the lobules is irrigated by the hepatic sinusoids, which are specialized capillaries organized around a vascular structure, i.e., a central vein that is connected with the other central veins of each lobule; together, these vascular structures end up constituting the major vascular system of the liver, the vena cava hepatic. The apical surface of the hepatocytes is in greater contact with the bile ducts, which produce the bile that flows into the duodenum. Thus, this particularly vascularized assembly ensures the very important blood flow of this organ.

One of the most original and unique properties of the liver tissue is its regenerative capacity. In the event of injury, this tissue regenerates in a process that takes between 1 and 2 weeks in humans, and becomes indistinguishable from the original tissue after a few weeks. The main factors that have been identified as being involved in this regenerative process are the hepatocyte growth factor (HGF), insulin, the transforming growth factor-alpha (TGFα), the epidermal growth factor (EGF), interleukin-6 (IL-6), and norepinephrine. Some of these factors and their receptors may have redundant roles depending on the species [7]. While the participation of tissue stem cells is undeniable in this regenerative process, the nature of these cells has been the subject of numerous studies and controversies [8, 9].

## Induced pluripotent stem cells, a source of hepatoblasts

The approaches that have been used to develop liver organoids are two-fold, as in many tissues. An organoid is defined as an in vitro three-dimensional structure that self-organizes from stem cells and has the capacity to self-renew and to differentiate to give rise to the different constitutive morphotypes of the tissue it aims to mimic and to reproduce at least some of its physiological functions. For liver organoids, there are protocols for obtaining spheroids/organoids from hepatic cell lines, such as HepG2 or HepaRG cells [10, 11], from biopsies or from already formed tissue [12–15]. In this first approach, the structures obtained depend on the quality of the samples, and their establishment requires an extracellular matrix and occurs over 4−8 weeks. The second approach is based on the use of pluripotent stem cells (PSCs). Adaptations of the protocols were made to allow the initial induction of those cells into a final endoderm, from which the hepatoblasts and then the hepatocyte-like cells are derived using a maturation process in the presence of various inducers and growth factors, but with a greater or lesser homogeneity in the cell types obtained [16–19]. The process that starts from PSCs is longer (10−15 weeks), but leads to a hepatic organoid structure that is more complex and closer to the functional level of liver tissue. The comparison of the two approaches revealed the advantages and disadvantages of each of the approaches [20].

Concomitantly, direct somatic reprogramming made it possible to obtain liver cells in both murine and human species using different combinations of genes, including *FOXA3*, *HNF1A*, and *HNF4A* [21, 22], or more complex systems, such as *HNF1A*, *HNF4A*, and *HNF6* supplemented with maturation factors (i.e., *ATF5*, *PROX1*, and *CEBPα*) [23]. A complementary and original approach consisted in the use of a single reprogramming factor (*HNF1α*) in the presence of different molecules that control specific signaling pathways [24]. This strategy resulted in the production of cells with markers and several hepatocyte functions in vivo.

If the cultures were initially developed into two-dimensional adherent cells, three-dimensional culture approaches have appeared more recently to allow the production of hepatic organoids and their long-term maturation. Thus, these recently described protocols allowed the establishment of structures that are maintained over time and for some of them reproduced “mature liver properties, including serum protein production, drug metabolism and detoxifying functions, active mitochondrial bioenergetics, and regenerative and inflammatory responses” by stably expressing several highly specific markers including ALB, SERPINA1, TTR, HNF4a, etc.… [25, 26]. The different steps of getting hepatocytes from PSCs are almost similar between the 2D and 3D cultures with the additional step of producing floating embryoid bodies for the 3D approach. The further steps of endoderm induction, endoderm specification, hepatoblast induction and hepatocyte maturation are more or less identical with changes in duration between the different reported protocols [19, 25, 26]. However, differences are indeed observed between both systems with advantages and disadvantages as summarized in Table 1. These same protocols are also starting to reproduce the complexity of the liver tissue, as mentioned above. In fact, this complexity supposes that all hepatocyte functions are ultimately represented in these derived structures in vitro, including the presence of vascularization and bile ducts [27–31]. Synthesis of the recent reported protocols is available [32].

**Table 1:** Comparison between 2 and 3D cultures for generating liver in vitro liver models

	2D culture	3D culture
Morphology	Monolayer	Organized aggregates with multiple layers
Amplification	Easy and convenient	More complex Stable long-term cultures
Differentiation	Directed but limited on flat and surface Constrained morphogenesis	Self-organization Feasibility of mixed 3D structures of hepatoblast, mesenchymal and vascular cells
Genes and Proteins	Hepatoblast and hepatocyte with embryonic phenotypes	More mature hepatocytes—adult-like phenotypes
Advantages	Fast and rapid establishment Relatively inexpensive, Well adapted to high throughput capacity	Partially mimicking the in vivo microenvironment Reproducing the apical-basal polarity in multicellular structures Control of factor gradient by microfluidic approaches
Disadvantages	Flat surface Automatic apical-basal polarity not mimicking the tissue structure	Costly and more laborious High throughput capacity to be optimized

Such analysis can be extrapolated for other organoid models as well.

The challenge of producing the most relevant hepatic organoids in relation to the hepatic tissue is particularly important for different approaches, such as the open perspective in regenerative medicine, but also as models for physiological studies of many hepatic pathologies, including viral infections such as hepatitis [33] and metabolic disorders such as fibrosis and cirrhosis that could both be due to chronic injuries, nonalcoholic fatty liver disease (NAFLD), the most prevalent chronic liver disease leading to the non-alcoholic steatosis (NASH), affecting millions of people around the world [34–36]. In this context, generation of hepatic stellate cells from PSC has been reported to also contribute to the modeling of liver fibrosis [37]. The hepatic organoid model is also becoming a reference in toxicology, because these structures appear to be a major advancement in the predictive approach to the evaluation of the toxicity of a known molecule or in therapeutic testing [38, 39].

## Applications in other species

Most of the studies and protocols published for generating organoids were performed using mouse and human models; thus, proportionally, the data available for other species, in particular species of agronomic interest, are scarce [40]. To highlight the interest of developing the hepatic organoid model in these species, it is also necessary to identify the similarities and the differences and therefore to compare the hepatic metabolisms between the different species, including humans. If the metabolic pathways are generally very close, the nutritional lever is also used in domestic animals, in particular to control carbohydrate metabolism and lipidogenesis which does not take place exclusively in the liver for mammals with fat deposits in peripheral tissues [41, 42].

Although the pig is often considered a possible alternative as an organ donor in xeno-transplantation approaches, few studies have been published on the formation of porcine hepatic organoids/spheroids [43], with rare exceptions, such as the bioartificial liver model that was developed for studying acute liver failure (ALF) that could be caused by various toxic compounds, chemicals or viruses that may induce a massive necrosis of parenchyma, responsible of ALF [44]. Conversely, the pig was also used as a test of the functionality of human liver organoids in this same ALF model [45]. However, there seems to be little use of this model in toxicological or nutritional approaches.

Another example is the production of hepatic organoids from tissue biopsies in cats, to propose a model for studying NASH [46]. As previously mentioned, modeling this disease is still a challenge [36] and even if numerous rodent models are developed as well as in vivo assays on minipigs, few in vitro assays with organoids of mammalian domestic animals were presently reported [42, 47, 48].

Various studies have also been carried out in the fish model, in particular for the generation of hepatic trout organoids in toxicological screening approaches [49–51].

All the current organoids obtained from species other than murine and human have been derived from tissue precursors, with PSCs and induced pluripotent stem cells (iPSCs) being neither described nor available in these species with the same properties as those observed in murine and human models. Recent advances in deriving PSCs in those species are summarized in the introductive chapter of this series, entitled “Organoids in domestic animals: with which stem cells?”. Therefore, despite those progresses, this obstacle of getting robust PSCs limits the development of original protocols in these species.

In avian species, the importance of lipid metabolism in the body composition, nutritional egg quality, and adaptation of birds to environmental changes such as feed composition changes [51–53], or during steatosis processes, render the liver an important tissue to be studied, as it is the major organ in lipid synthesis in contrast to mammals [41, 42]. As an example and as stated by Surugihalli et al., “the chicken liver is subjected to intense lipid burden from high rates of yolk-lipid oxidation and also from the accumulation of the yolk-derived and newly synthesized lipids from carbohydrates”. High rates of hepatic lipid oxidation and lipogenesis are also central features of non-alcoholic fatty liver disease (NAFLD) in both rodents and humans, but is associated with impaired insulin signaling, dysfunctional mitochondrial energetics and oxidative stress” [54]. Numerous studies have investigated the molecular basis of the avian hepatic metabolism by deciphering mainly the hepatic lipid metabolism and the associated genes [55–57]. In this context, organoids in avian species would be of great interest for studying the regulatory mechanisms of lipid metabolism. Furthermore, chicken has diverged from mammals more than 300 M years ago and is widely used to evaluate the level of gene conservation between species during evolution, with this conservation being a sign of a major biological role. Examples of lncRNA have been also reported for liver and adipose tissue as well [58].

**Figure 1:**
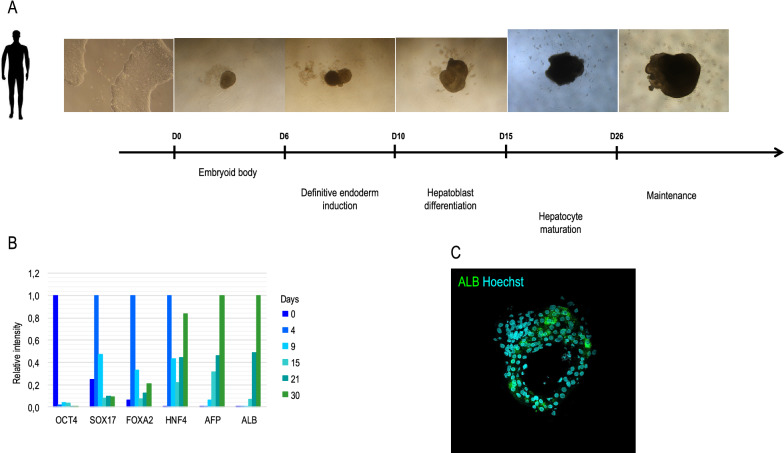
**A schematic description of the generation of liver organoïds from hiPSC. A** hiPSC were induced into definitive endoderm before being turned into hepatoblast and more mature hepatocyte following an adaptation of the Rashidi et al., protocol [22]. **B** The expression of different markers was detected by qRT-PCR and illustrates the loss of pluripotent marker (OCT4), the appearance of endoderm ones (SOX17, FOXA2) and then more specific hepatic markers (HNF4, AFP, ALB). **C** On mature organoïds, Albumin (ALB) is detectable by immunocytochemistry.

**Figure 2:**
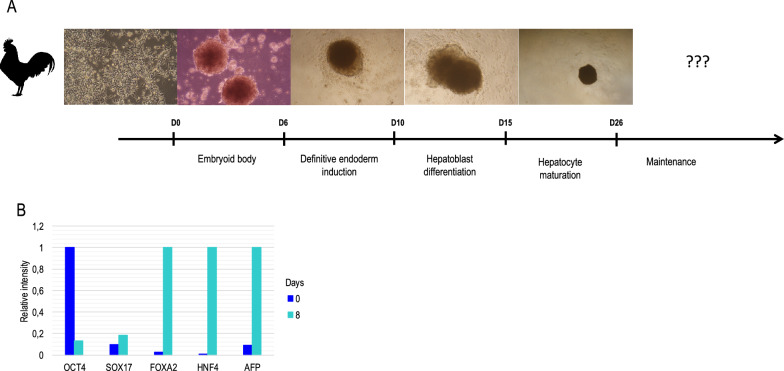
**Preliminary data with chicken liver organoïds derived from chicken embryonic stem cells (cESC). A** cESC were induced into endoderm before being turned into hepatoblasts, but failed to be induced in more mature hepatocytes under present tested protocols. **B** the expression of different markers was detected by qRT-PCR and illustrates the loss of pluripotent marker (OCT4), the weak induction into endoderm (SOX17) and then the present failure to get more specific hepatic markers (FOXA2, HNF4, AFP) with the current conditions.

In avian species, few data are available at the level of the preparation of hepatocytes and their maintenance in culture for several days, but in each case, they were derived from adult tissue [59–62]. However, the development of organoids in avian species has not been reported. As embryonic stem cells were obtained and described in chicken [63–65], we initiated the induction of the differentiation of these PSCs in the hepatic pathway based on the human and murine models. Validated in the human model with hiPSCs, we developed a protocol that allows the establishment of hepatic organoids that produce albumin (Figure 1). In a preliminary study, this same protocol does not seem to be compatible with bird PSCs and no long-term liver organoids were obtained, even if hepatic markers were weakly detected (Figure 2). Therefore, adaptations of each step have been carried out, starting from the formation of cell aggregates that are transferred into an endodermic induction medium. These structures exhibited a pattern of proliferation and induction of markers that is specific to the engagement into this lineage. Additional work is needed.

## Conclusion

The development of hepatic organoids represents a real challenge for mimicking the complexity of hepatic tissue and its multiple physiological functions. Additional challenges remain to be overcome for domestic species, including the plasticity of pluripotent stem cells and the adaptation of the protocols currently described and used in the human model. We can hope that significant progress will be made in the years to come to have such in vitro models in these species.

## Abbreviations

ALF: Acute liver failure
BMPs: Bone Morphogenetic Factors
EGF: Epidermal Growth Factor
FGF: Fibroblast Growth Factor
HGF: Hepatocyte Growth Factor
IL-6: Interleukin-6
iPSC: Induced pluripotent stem cell
NAFLD: Nonalcoholic fatty liver disease
NASH: Non-alcoholic steato-hepatitis
PSC: Pluripotent Stem Cell
TGFα: Transforming Growth Factor alpha
